# An in-vitro exploration of the antifibrotic activity of Naringenin: A potential therapeutic agent for oral submucous fibrosis management

**DOI:** 10.1016/j.jobcr.2025.06.006

**Published:** 2025-06-12

**Authors:** S. Samyuktha Aarthi, Deepak Pandiar, Raghunandhakumar Subramanian, Reshma Poothakulath Krishnan

**Affiliations:** a Department of Oral Pathology and Microbiology, Saveetha Dental College and Hospitals, Saveetha Institute of Medical and Technical Sciences, Saveetha University, Chennai, Tamil Nadu, India; b Cancer and Stem Cell Research Lab, Department of Pharmacology, Saveetha Dental College and Hospitals, Saveetha Institute of Medical and Technical Sciences, Saveetha University, Chennai, Tamil Nadu, India

**Keywords:** Naringenin, Oral submucous fibrosis, Arecoline, Human gingival fibroblasts, TGF-β, Fibrosis, Antifibrotic therapy

## Abstract

**Background:**

Oral submucous fibrosis (OSMF) is a precancerous condition primarily associated with betel nut chewing. Naringenin, a flavonoid found in citrus fruits, has been demonstrated to show antifibrotic effects in various fibrosis models. The present study was conducted to investigate the potential antifibrotic properties of naringenin in Human gingival fibroblasts (HGFs) exposed to arecoline.

**Materials and methods:**

Naringenin was extracted from grapefruit peel using methanol and characterized via Gas Chromatography-Mass Spectrometry (GC-MS). HGFs were cultured in Dulbecco's Modified Eagle Medium and treated with arecoline to induce fibrosis. The cells were then exposed to naringenin at varying concentrations. Cytotoxicity was assessed using the MTT assay, while the expression of fibrotic markers was quantified using real-time polymerase chain reaction (PCR). Additionally, Masson's trichrome staining was performed to evaluate the collagen deposition aided by An in-silico pharmacological network analysis.

**Results:**

GC-MS confirmed the presence of naringenin as a major bioactive compound in grapefruit peel extract. Naringenin significantly improved cell viability in arecoline-treated HGFs. It was found that naringenin markedly downregulated the expression of fibrotic markers, as compared to the arecoline-only group. Histopathological analysis demonstrated a reduction in collagen deposition following naringenin treatment. Pharmacological network analysis identified potential pathways targeted by naringenin, including TGF-β, PI3K-Akt, and MAPK signaling, with hub genes such as MMP9 and TGFB1 playing central roles.

**Conclusion:**

Naringenin exhibits promising antifibrotic activity in arecoline-induced fibrosis in HGFs, potentially through modulation of key fibrotic signaling pathways. These findings highlight its potential role as a therapeutic agent for OSMF management.

## 1 Introduction

Nearly half of deaths in affluent nations are caused by fibrosis, which is the final common pathology of most chronic diseases and can be found in the skin, kidney, liver, lung, and heart.^1^ Although there are many different causes of fibrosis in the different organs, the most prevalent etiologies are genetic changes, age, and inflammation. Type I collagen (COL1), type III collagen (COL3), and fibronectin (FN) are just a few of the extracellular matrix (ECM) proteins that are heavily deposited during fibrosis, along with the generation of inflammatory agents and fibroblast activation and proliferation.^2^ A major concern with OSMF is its malignant transformation potential, particularly into oral squamous cell carcinoma (OSCC).^3^^,^^4^ Despite significant advancements in the study of organ fibrosis over the past few decades and a growing understanding of the onset and progression of fibrosis, there is currently no proven cure for fibrotic disorders.^5^

A vast majority of the cases of OSMF have been reported from Southeast Asian nations such as India, Pakistan, Taiwan.^6^ The primary ingredients in betel quid are arecoline from betel nuts and copper, which induces fibroblast dysfunction and the formation of fibrotic bands.^7^ The main characteristic of OSMF, it affects much of the oral cavity and causes advanced lockjaw because of stiffness in the cheeks, lips, pharynx, and upper part of the oesophageal canal. This condition eventually leads to dysphagia.^8^ There are various treatment modalities available for managing potentially malignant oral disorders, including corticosteroids, epigenetic modulators and mesenchymal stem cell therapy.^9^^,^^10^ However, there is a growing demand for natural bioactive compounds as individuals increasingly prioritize health and wellness, particularly in connection with health-promoting diets. Epidemiological studies have consistently suggested that a high dietary intake of phytochemicals, especially polyphenols, is associated with a reduced risk of various chronic diseases.^11^

Global citrus production, including oranges, mandarins, lemons, bergamots, limes, pummelos, and grapefruits, has witnessed significant growth in recent decades, surpassing 100 million metric tons annually. Approximately one-third of citrus fruits are processed for fresh juice or citrus-based beverages, with the juice yield accounting for only half of the fruit's weight. This results in a substantial accumulation of peel and pulp waste on a global scale.^12^ Importantly, research has identified citrus peels as a major source of polyphenols, including phenolic acids, flavonoids, polymethoxyflavones (PMFs), flavanones, and glycosylated flavanones. Previous studies have focused on the antifibrotic potential of citrus-derived compounds, particularly those extracted from grapefruit pulp and juices, particularly naringenin, however, its role in the management of fibrosis in the oral cavity has not been explored so far.^13^, ^14^, ^15^ Naringenin is preferred over other flavonoids in the treatment of oral submucous fibrosis (OSMF) due to its superior antioxidant, antifibrotic, and anti-inflammatory properties. It effectively scavenges reactive oxygen species (ROS), thereby mitigating oxidative stress, a key factor in arecoline-induced fibrogenesis. Compared to other flavonoids, naringenin more potently downregulates fibrogenic markers such as TGF-β1, α-SMA, and collagen I, which are central to the progression of OSMF. Additionally, it inhibits pro-fibrotic pathways like TGF-β/Smad and inflammatory mediators including NF-κB, IL-6, and TNF-α.^16^, ^17^, ^18^ Its relatively better bioavailability and tissue penetration further enhance its therapeutic potential, making it a promising candidate among citrus bioactives for managing OSMF. Application of grapefruit peel not only promotes sustainable waste management but also expands the scope of citrus-based therapies. By highlighting the therapeutic relevance and environmental sustainability of utilizing grapefruit peel, a wasteful byproduct, this study not only offers a potential natural remedy for OSMF but also contributes to the advancement of green pharmacology.^19^ While previous studies have explored the general antioxidant and anti-inflammatory properties of citrus flavonoids, the specific antifibrotic effect of grapefruit peel-derived naringenin in the context of arecoline-induced fibrosis in HGFs remains under-investigated. Most existing research focuses on extracts derived from pulp or juice, overlooking the therapeutic potential of peel-based bioactives, which are typically discarded as agro-waste.^20^

Specifically, the study seeks to assess the effect of naringenin on the fibrotic markers in arecoline-stimulated human gingival fibroblasts using in vitro assays, alongside exploring the underlying signaling pathways modulated by naringenin through in silico molecular docking and pathway enrichment analysis. Therefore, this study aims to assess the antifibrotic potential of naringenin, isolated from grapefruit peel, using an in vitro arecoline-induced fibrosis model in HGFs. By integrating in vitro assays with in silico pharmacological network analysis, the study further seeks to elucidate the key molecular pathways modulated by naringenin. This dual approach not only provides mechanistic insight but also highlights the translational relevance of naringenin as a cost-effective, natural therapeutic alternative for OSMF. Additionally, utilizing grapefruit peel—a common agro-waste—aligns the research with principles of sustainability and waste valorization. We hypothesize that naringenin, isolated from grapefruit peel, can prevent arecoline-induced fibrosis in human gingival fibroblasts (HGFs) by downregulating key fibrotic markers and modulating associated signaling pathways involved in fibrosis progression.

## 2 Materials and methods

### 2.1 In vitro study design

After obtaining clearance from the institutional scientific review board (SRB/SDC/OPATH-2305/24/163) and human ethical clearance committee (IHEC/SDC/OPATH-2305/24/149), an in-vitro study was conducted to investigate the antifibrotic effects of bioactive compounds identified in the grapefruit peel extract on OSMF. HGFs were used as the cellular model, given their relevance in extracellular matrix deposition and fibrosis. After obtaining the consent, HGFs were isolated from the gingival tissue of normal adults during third molar extraction or gingivectomy (aged from 18 to 25 years). The isolation was performed by enzymatic digestion, subjected to collagenase (900 u/mL) and dispase (400 u/mL) digestion at 37 °C for 1 h. The cell cultures were performed with RPMI 1640 (Invitrogen Corporation, CA, USA) supplemented with 20 % (v/v) fetal bovine serum, 100 U/mL penicillin, and 100 μg/mL streptomycin at 37 °C with 5 % CO_2_. The culture medium was changed every three days and sub-cultured at 80 % confluence. At passage 2, cells were seeded in culture dishes in all in vitro experiments for this study. Specifically, cells were maintained in a humidified incubator set at 95 % humidity and 5 % CO_2_ at 37 °C. All experimental procedures were carried out using cells between passages 4 and 7. Treatments were performed when cells reached approximately 70–80 % confluency to ensure uniform cell behavior and minimize senescence-related variability.

Arecoline was employed to induce fibrosis in HGFs, mimicking the pathogenic processes of OSMF. Arecoline hydrobromide was obtained from a reputable chemical supplier (Sigma-Aldrich Chemicals Private Limited, Bangalore, India), with a purity of >98 %. It was dissolved in sterile distilled water for stock preparation and filtered using a 0.22 μm syringe filter to ensure sterility. Dilutions were freshly prepared for each treatment. The specific concentrations tested in cytotoxicity and fibrosis-induction assays were estimated at different concentration (e.g., 25, 50, 100, 150, and 200 μg/mL), along with the rationale for IC_50_-based selection. Table 1 shows the detailed information of the concentration of arecoline and naringenin along with treatment duration, and sample size.

**Table 1:** Details of the concentration of arecoline and naringenin along with treatment duration and sample size.

Group	Treatment Description	Arecoline (μg/mL)	Naringenin (μM)	Treatment Duration	Sample Size
Control	Untreated	0	0	24, 48, 72 h	n = 3
Arecoline only	Arecoline dose range	5–200	–	24 h (MTT assay)	n = 3
Grapefruit Extract	Methanolic extract of peel	20–300	–	24 h (MTT assay)	n = 3
Naringenin only	Naringenin dose range	–	10–200	24 h (MTT assay)	n = 3
Arc-treated	Arecoline at IC_50_ dose	50	–	48 h (qPCR/IF)	n = 3
Arc + Naringenin	Arecoline + Naringenin co-treatment	50	40	48–72 h (qPCR/IF)	n = 3

### 2.2 Extraction

Fresh grapefruit peels (Citrus x paradisi L.) were procured from the local market in Chennai, sliced, and manually separated into albedo and flavedo layers. Grapefruit peels were dried, powdered, and subjected to maceration in methanol (HPLC grade, 99.9 %) at room temperature for 72 h under continuous agitation. The extract was filtered and concentrated under reduced pressure using a rotary evaporator. No further purification was performed; thus, the extract was used in its crude form.

### 2.3 Characterization of metabolites by gas chromatography/Mass spectroscopy (GC-MS)

Following filtration, the crude extract was analyzed using Gas Chromatography-Mass Spectrometry (GC-MS-QP2010 Plus system - Shimadzu, Japan) to identify key metabolites. The methanolic extract was filtered through a 0.45 μm membrane and analyzed using a GC-MS system equipped with an HP-5MS non-polar column. The carrier gas used was helium at a flow rate of 1 mL/min, with the injector temperature set to 250 °C. The oven temperature was programmed to start at 50 °C, then ramp up at 10 °C per minute to 250 °C, where it was held for 10 min. The mass spectrometer operated in electron ionization (EI) mode at 70 eV, scanning a mass range of *m/z* 50–550. The compounds were identified by their retention times and fragmentation patterns compared with NIST and Wiley libraries.

### 2.4 Dose selection of naringenin

The 40 μg/mL concentration of naringenin was selected based on preliminary MTT results indicating significant antifibrotic activity without cytotoxicity. This dose also corresponded to an effective range observed in earlier studies on similar fibroblast models.

### 2.5 Fibrosis induction

HGF were cultured in high-glucose Dulbecco's Modified Eagle Medium, supplemented with 10 % fetal bovine serum (FBS) and 1 % penicillin-streptomycin. Cells were seeded at an optimal density in culture plates and incubated under controlled conditions. Arecoline was introduced at concentrations ranging from 0 to 200 μg/ml, based on IC50 values determined in preliminary dose-response experiments. Treatment durations ranged from 24 to 72 h, during which morphological changes and proliferation rates were monitored to confirm fibrosis induction. These time points were chosen based on preliminary observations that fibrosis markers begin to show significant upregulation after 24 h of arecoline exposure and plateau by 72 h. Untreated cells served as negative controls to validate experimental results.

### 2.6 Cytotoxicity assay

The MTT assay was conducted to assess the cytotoxic effects of arecoline, grapefruit crude extract, and naringenin on human gingival fibroblasts (HGFs). Cells were seeded in 96-well plates at a density of 1 × 10^5^ cells/well and allowed to adhere overnight at 37 °C. The experimental groups were treated with varying concentrations (0–200 μg/mL) of the test compounds, while untreated wells served as the control. Following incubation, 100 μL of MTT reagent (10 mg/mL) was added to each well and incubated for 3–4 h to facilitate the formation of formazan crystals by metabolically active cells. Subsequently, the medium was replaced with 100 μL of dimethyl sulfoxide (DMSO) to solubilize the crystals, and absorbance was measured at 570 nm using a Synergy H1 Hybrid Multi-Mode microplate reader.

### 2.7 Quantitative PCR (qPCR) analysis of fibrotic markers

To evaluate the anti-fibrotic effects of naringenin, quantitative PCR (qPCR) was performed to assess the expression levels of key fibrotic markers, including collagen type I (Col-1), α-smooth muscle actin (α-SMA), and transforming growth factor-beta (TGF-β). Human gingival fibroblasts (HGFs) were treated with arecoline alone and in combination with naringenin, with untreated cells serving as the control group (Table 2). All in vitro assays, including MTT, qPCR, and staining procedures, were performed in triplicate (n = 3 biological replicates), and each biological replicate included three technical repeats.

**Table 2:** Relative gene expression of fibrotic markers analyzed by qPCR; SD−standard deviation.

Marker	Group	Mean ± SD	Fold Change (vs. Control)	p-Value
α-SMA	Control	1.00 ± 0.00	1	0
	Arecoline treated	1.89 ± 0.39	1.89	0.015
	Arecoline + Naringenin	0.78 ± 0.52	0.78	0.013
COL-type1	Control	1.00 ± 0.00	1	0
	Arecoline treated	1.76 ± 0.48	1.76	0.03
	Arecoline + Naringenin	0.62 ± 0.21	0.62	0.025
TGF-β	Control	1.00 ± 0.00	1	0
	Arecoline treated	1.66 ± 0.58	1.66	0.054
	Arecoline + Naringenin	0.45 ± 0.18	0.45	0.024

Total RNA was extracted using the TRIzol reagent and quantified using a NanoDrop spectrophotometer. Reverse transcription was carried out using a cDNA synthesis kit, and gene-specific primers were designed for qPCR amplification. The following primer sequences were used for quantitative PCR analysis: TGF-β1 forward: 5′-TCCTGGCGATAACCTCAGCAA-3′, TGF-β1 reverse: 5′-CTCAATTTCCCCTCCACGGGC-3′; α-SMA forward: 5′-GTCCCAGACATCAGGGAGTAA-3′, α-SMA reverse: 5′-TCGGATACTTCAGCGTCAGGA-3′; COL1A1 forward: 5′-ATGGATTCCAGTTCGAGTAGGC-3′, COL1A1 reverse: 5′-CATCGACAGTGACGCTGTAGG-3′; GAPDH forward: 5′-CTACTGGCGCTGCCAAGGCTGT-3′, and GAPDH reverse: 5′-GCCATGAGGTCCACCACCCTGT-3′. qPCR was performed using SYBR Green Master Mix (Applied Biosystems, USA) on the StepOnePlus Real-Time PCR System under the following conditions: initial denaturation at 95 °C for 5 min, followed by 35 cycles of 95 °C for 40 s, gene-specific annealing temperatures (58–60 °C) for 30 s, and extension at 72 °C for 1 min, with a final extension at 72 °C for 15 min. Melt curve analysis was conducted to confirm specificity of amplification, and all primer pairs exhibited single peaks. Amplification efficiency for each primer set ranged between 90 and 110 %. GAPDH was selected as the reference gene after comparative stability analysis with β-actin using geNorm software, which confirmed its consistent expression across all treatment conditions. Each reaction was run in triplicate with three biological replicates, and relative expression levels were calculated using the 2^−ΔΔCt method.

### 2.8 Histopathological analysis using Masson's trichrome staining

To evaluate collagen deposition in primary buccal mucosal fibroblasts, Masson's trichrome staining was performed on control, arecoline-treated (disease control), and naringenin-treated (40 μg/mL post-arecoline induction) groups. Negative and positive controls were used during each run. Cells were cultured under standard conditions (37 °C, 5 % CO_2_) and fixed with 4 % paraformaldehyde after 48 h of treatment. Staining was carried out using a standard Masson's trichrome protocol, wherein nuclei were stained with hematoxylin, cytoplasm and muscle fibers with acid fuchsin and xylidine ponceau, and collagen fibers with aniline blue. After sequential dehydration, slides were mounted and examined using a bright-field light microscope (Olympus CX43) at 40 × and 100 × magnifications. Images were captured using a ToupTek digital camera with ToupView software, and no post-processing (e.g., contrast enhancement or filtering) was performed to preserve image authenticity.

### 2.9 In silico pharmacological network analysis

The pharmacological potential of naringenin was analyzed computationally to identify its molecular targets and associated pathways in OSMF. Naringenin's potential targets were retrieved from the Swiss Target Prediction database, while disease-related genes were sourced from GeneCards and OMIM. A Venn diagram analysis identified 27 overlapping targets, which were subjected to Gene Ontology (GO) and KEGG enrichment analyses. Pathways such as TGF-β, ECM-receptor interaction, PI3K-Akt signaling, and proteoglycan pathways were significantly enriched. Protein-protein interaction (PPI) networks, constructed using the STRING database, identified hub genes, including MMP2, MMP9, and TGFB1, with a confidence score threshold of >0.7 (high confidence). These genes were central to naringenin's antifibrotic effects, further validated by molecular docking studies (Table 3).

**Table 3:** Depiction of Key Hub Genes, Interaction Degree, and Associated Pathways used for In silico analysis.

HUB GENES	Degree of interaction	Biological pathway
BCL2	13	Apoptosis regulation via mitochondrial outer membrane permeabilization and inhibition of caspase activation.
COL18A1	11	Inhibition of angiogenesis and extracellular matrix (ECM) organization via endostatin-mediated VEGF signaling suppression.
ESR1	10	Estrogen signaling pathway regulating gene transcription, cell proliferation, and differentiation.
IGFBP3	13	IGF signaling modulation and IGF-independent apoptosis via IGFBP-3 receptor (TMEM219).
KDR	13	VEGF signaling pathway mediating angiogenesis, endothelial cell proliferation, and migration.
MMP1	13	ECM remodeling through degradation of interstitial collagens (types I, II, III).
MMP2	14	ECM degradation and tissue remodeling via type IV collagen breakdown.
MMP3	14	ECM degradation and wound healing by breaking down fibronectin and proteoglycans.
MMP9	14	ECM remodeling and leukocyte migration via type IV/V collagen and fibronectin degradation.
PLG	13	Fibrinolysis and tissue remodeling via conversion to plasmin, degrading fibrin and ECM components.
SERPINE 1	14	Regulation of fibrinolysis by inhibiting tissue and urokinase-type plasminogen activators (PLAT/PLAU).
TGFB1	14	TGF-β signaling pathway regulating fibrosis, immune responses, and ECM production
TIMP 1	13	Inhibition of MMP activity to regulate ECM turnover and prevent excessive tissue degradation.
TIMP 2	13	Inhibition of MMPs (e.g., MMP-1, MMP-2) to maintain ECM homeostasis.
TIMP 3	14	ECM stabilization via inhibition of multiple MMPs and participation in acute tissue response

### 2.10 Statistical analysis

All statistical analyses were conducted using GraphPad Prism (version 10.4.2). One-way ANOVA followed by Tukey's post hoc test was employed to determine statistical significance across multiple groups. A p-value of <0.05 was considered statistically significant. Data were normalized to the untreated control group for consistency across experiments. Outlier detection was performed using Grubbs' test, and identified outliers were excluded based on standard statistical justification.

## 3 Results

### 3.1 Chemical components of methanolic crude grape fruit extract by GC-MS analysis

**Fig. 1:**
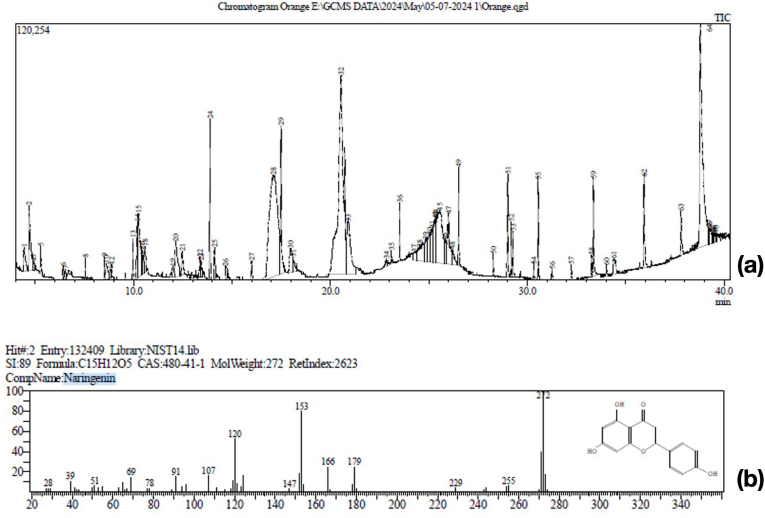
Graph demonstrating characterization of metabolites present in the grapefruit extract using Gas Chromatography/Mass Spectroscopy (GC-MS).

GC-MS analysis of the methanolic extract of grapefruit peel revealed a range of volatile and semi-volatile phytochemicals. The Total Ion Chromatogram (TIC) displayed multiple distinct peaks, indicating a diverse composition of secondary metabolites (Fig. 1a). Among the identified compounds, Naringenin was detected as a predominant component, with a retention time consistent with its known profile. The molecular formula was determined as C_15_H_12_O_5_, with a molecular weight of 272 g/mol and a similarity index of 89 when compared with the NIST14 library (Fig. 1b). Its fragmentation pattern corresponded well with reference spectra, confirming its structural identity.

### 3.2 Cell viability

**Fig. 2:**
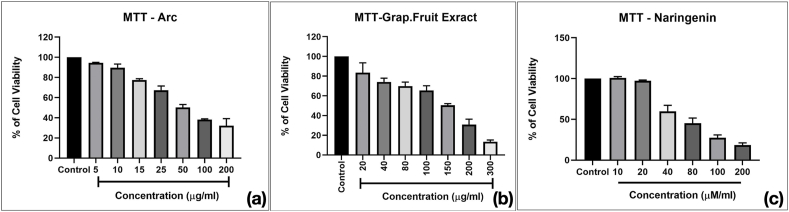
Graphs demonstrating the comparative cell viability using the MTT assay to determine the viability in a dose-dependent manner for arecoline (a), grapefruit crude extract (b) and naringenin (c). Data are represented in mean ± SD of results from three independent experiments. Half-maximal inhibitory concentration (IC50) for arecoline was determined as 50 g/mL, for methanolic grape Fruit crude extract as 150 μg/mL, and for Naringenin 40 g/mL.

**Fig. 3:**
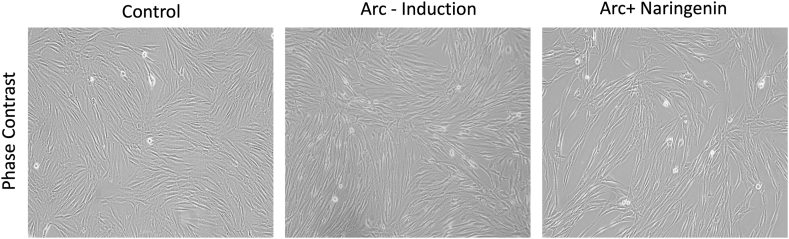
Morphological evaluation of primary buccal mucosal fibroblast using phase contrast microscopy. Control, disease control (arecoline treated) and treatment with Naringenin 40 μg/mL (10x).

The results of the MTT assay demonstrate the effects of arecoline, methanolic grape fruit crude extract, and Naringenin on the viability of buccal mucosal fibroblasts at varying concentrations. As shown in the graphs, all three treatments led to a dose-dependent reduction in cell viability. Arecoline exhibited an IC50 value of 50 μg/mL, indicating significant cytotoxicity at lower concentrations. The methanolic grape fruit crude extract showed moderate cytotoxic effects with an IC50 of 150 μg/mL, whereas Naringenin displayed the highest potency with an IC50 of 40 μg/mL. The IC50 values were determined for each compound, revealing that naringenin exhibited the highest cytotoxic potency with an IC50 of 40 μg/mL, followed by grapefruit crude extract (IC50 = 150 μg/mL). Comparatively, arecoline displayed moderate cytotoxicity. These findings, illustrated in Fig. 2, suggest that naringenin significantly inhibits fibroblast viability, demonstrating its potential therapeutic efficacy over arecoline and grapefruit extract. The control fibroblasts exhibited a normal, elongated spindle shape, typical of healthy fibroblast morphology. The cells are evenly distributed, with intact cellular structures and no visible abnormalities. The Arc-Induction (Arecoline-treated) fibroblasts showed altered morphology, with increased cell density and disorganized arrangement. There was an apparent loss of the typical spindle shape, indicating the induction of dense fibrotic changes by arecoline. This reflects cellular stress and a potential shift towards myofibroblast differentiation. Arc + Naringenin treated cells [with naringenin (40 μg/mL)] demonstrated partial restoration of normal morphology. Fibroblasts appeared less dense, with a return towards the spindle shape and organized arrangement, suggesting the antifibrotic effect of naringenin in counteracting arecoline-induced morphological changes (Fig. 3).

### 3.3 Fibrotic markers analyses

The qPCR analysis reveals that the expression of fibrotic markers α-SMA, Col-type1, and TGF-β is significantly elevated in the Arc-treated group compared to the control, indicating increased fibrosis and ECM deposition. However, treatment with Naringenin extract (Arc + Naringenin extract) notably reduced the expression of these markers compared to the Arc-treated group. Specifically, α-SMA expression, associated with myofibroblast activity, and Col-type1, indicative of collagen synthesis, are both significantly downregulated, demonstrating Naringenin's ability to mitigate fibrosis. Similarly, the marked reduction in TGF-β expression in the Naringenin-treated group highlights its role in attenuating profibrotic signaling pathways.

**Fig. 4:**
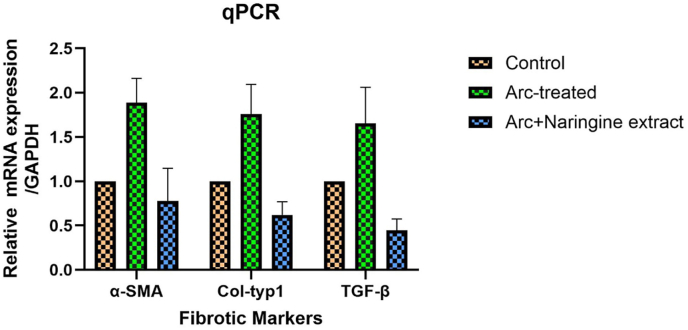
qPCR analyses revealed a significant decrease in the expression of collagen type I, α-SMA, and TGF-β in cells treated with naringenin compared to those treated with arecoline alone.

As illustrated in Fig. 4, treatment with naringenin significantly reduced the expression of fibrotic markers compared to arecoline-treated cells. Specifically, a marked decrease in α-SMA, collagen type I, and TGF-β expression levels was observed in cells co-treated with naringenin, suggesting its potential in mitigating arecoline-induced fibrosis. These findings reinforce naringenin's therapeutic potential in oral submucous fibrosis (OSMF) by downregulating key profibrotic pathways.

### 3.4 Histopathological analyses

**Fig. 5:**
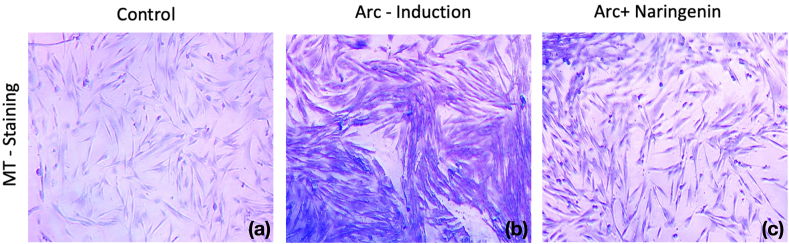
The morphological evaluation of primary buccal mucosal fibroblasts was conducted using Masson's trichrome staining to compare collagen production qualitatively. The study included three groups: control, disease control (arecoline-treated), and naringenin-treated (40 μg/mL) (10x).

As shown in Fig. 5, arecoline-treated fibroblasts exhibited dense collagen deposition, indicating fibrosis. However, in the naringenin-treated group, collagen accumulation was significantly reduced, resembling the morphology of control fibroblasts. These findings suggest that naringenin attenuates arecoline-induced fibrosis by reducing excessive collagen synthesis, highlighting its potential as an anti-fibrotic agent.

### 3.5 Establishment of a pharmacology network with naringenin for OSMF target analysis

The pharmacology network analysis of naringenin for oral submucous fibrosis (OSMF) revealed significant insights into its antifibrotic mechanisms. A total of 77 potential targets for Naringenin and 1138 OSMF-related targets were identified. Using Venn diagram analysis, 27 common targets were found, representing the intersection between naringenin-associated and OSMF-related genes (Fig. 5a). These shared targets were considered key candidates for further investigation into the antifibrotic activity of naringenin.

Gene Ontology (GO) and KEGG enrichment analyses of the 27 overlapping targets highlighted significant molecular pathways and biological processes involved in OSMF pathogenesis. The KEGG pathway analysis (Fig. 5b) identified critical signaling pathways such as ECM-receptor interaction, PI3K-Akt signaling, and proteoglycans in cancer, emphasizing their relevance to tissue remodeling and fibrosis. The GO enrichment analysis (Fig. 5c) revealed biological processes like extracellular matrix organization and collagen metabolic processes, molecular functions such as collagen binding, and cellular components related to the extracellular matrix and basement membrane.

**Fig. 6:**
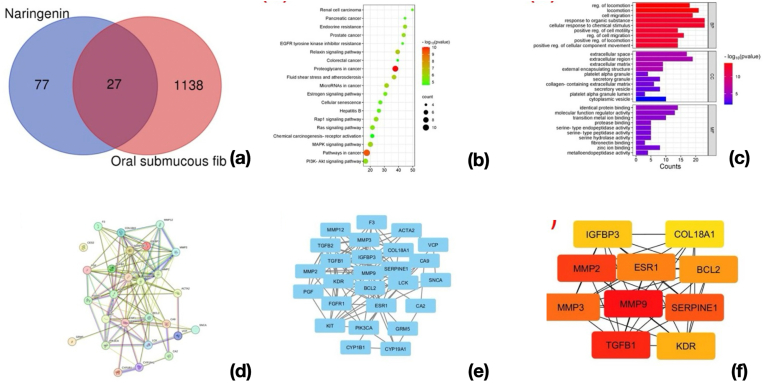
Image showing the results from an in-silico study involving naringenin, specifically related to its effects on oral submucous fibrosis (OSMF). 6a) Venn diagram, 6b) KEGG pathway analysis, 6c) GO enrichment analysis, 6d) STRING, 6e-f) Subnetwork analyses.

To further explore molecular interactions, a protein-protein interaction (PPI) network was constructed using STRING (Fig. 6d). The PPI network highlighted the connectivity among key targets, with the size of the nodes representing their interaction degree. Subsequent subnetwork analyses (Fig. 6e–f) identified hub genes, including MMP2, MMP3, MMP9, TGFB1, and ESR1, as central regulators of fibrosis-related processes. These hub genes are involved in extracellular matrix degradation, inflammation regulation, and tissue remodeling, underscoring their importance in the antifibrotic activity of naringenin. Overall, this integrated analysis demonstrates that naringenin targets critical pathways and genes associated with OSMF, offering a molecular basis for its therapeutic potential, supporting the hypothetical mechanism proposed in the present study.

## 4 Discussion

A wide variety of diseases are seen affecting the oral cavity modulated by various systemic conditions and may have a contributing role of environmental conditions and genetics.^21^ Oral submucous fibrosis is one such disease, which has a strong correlation with the intake of areca nut and tobacco products.^22^ The study demonstrates that naringenin exerts significant antifibrotic effects on arecoline-induced fibrosis in human gingival fibroblasts, a potential therapeutic agent for oral submucous fibrosis (OSMF). By effectively reducing the expression of key fibrotic markers and decreasing collagen deposition, naringenin appears capable of modulating the fibrotic process. The mechanism behind this effect is likely related to the inhibition of the TGF-β signaling pathway, which plays a central role in the pathogenesis of OSMF.

In the present study, for the first time, we employed the used and displayed the role of naringenin, derived from grapefruit peel, in the management of OSMF. Peel valorization may be used as a sustainable, low-cost bioresource that supports the circular bioeconomy. The use of grapefruit peel as a novel, sustainable, and underutilized natural source of bioactive compounds aligns with principles of green chemistry and resource optimization, making it particularly relevant for application in low-resource settings. We adopted an integrative methodological approach, combining in vitro cellular assays with in silico molecular docking to provide both empirical and predictive insights into the antifibrotic potential of naringenin. Standardized and reproducible techniques, such as GC-MS for compound identification and quantitative PCR for gene expression analysis, confer methodological rigor and facilitate comparability with other studies. Furthermore, the in silico mechanistic predictions corroborated the functional outcomes observed in vitro, strengthening the evidence for naringenin's biological activity. Lastly, given the increasing burden of oral submucous fibrosis (OSMF) in populations with limited access to advanced healthcare, the identification of a naturally derived, potentially affordable therapeutic candidate highlights the public health significance of this research.

Naringenin has been identified in various parts of grapefruit using different analytical approaches, reflecting its distribution across fruit matrices. A previous study on pink grapefruit pulp (Citrus × paradisi Macfad) identified naringenin using chromatographic analysis at 280 nm, detecting it at a retention time of 6.94 min, corresponding to the specific chromatographic region for this compound.^16^ In contrast, our study focused on the methanolic extract of grapefruit peel analyzed by GC-MS, where naringenin was identified as a major constituent with a retention time consistent with its molecular formula (C_15_H_12_O_5_), molecular weight (272 g/mol), and a similarity index (SI) of 89 based on the NIST14 library. While the earlier study demonstrated the presence of naringenin in the pulp, our findings highlight its significant concentration in the peel, supported by its prominent peak in the GC-MS chromatogram.

In a previous study, it was demonstrated that naringenin bears a significant anticancer potential against breast cancer cell lines by inhibition of proliferation rate in a dose and time dependent manner.^23^ While, Ahamed et al. reported similar effects in epidermoid carcinoma A431 cells, showing significant inhibition of cell proliferation and morphological changes such as shrinkage and detachment.^24^ In our study, the MTT assay revealed that naringenin improved the viability of arecoline-treated human gingival fibroblasts (HGFs) in a dose-dependent manner, indicating its protective role against cytotoxicity. These findings highlight naringenin's dual potential in reducing cancer cell viability and restoring normal cellular function under toxic conditions, emphasizing its broader therapeutic applications. Our study also found that naringenin exhibited concentration-dependent cytotoxicity at higher doses, as demonstrated by the MTT assay. This duality underscores the importance of defining an optimal therapeutic window, balancing efficacy with safety, in future investigations.

Supporting studies reinforce this potential mechanism. In an in vitro study by Liu et al., naringenin was identified as a Smad3-specific inhibitor, capable of suppressing TGF-β1-induced extracellular matrix (ECM) protein expression in cultured rat hepatic stellate cells (HSCs). This inhibition occurred via selective blocking of Smad3 activation, a downstream target in the TGF-β pathway, suggesting that naringenin can effectively interfere with TGF-β-mediated fibrotic signaling.^25^ As discussed previously naringenin treatment reduced hepatic inflammation, liver fibrosis, and hepatocyte apoptosis, thereby improving liver function. This was achieved by lowering the levels of NF-κB, TGF-β1, and caspase-3, which are all involved in fibrosis and inflammatory responses, further supporting naringenin's role as an antifibrotic agent.^26^ Similarly, in this study, naringenin significantly improved the viability of arecoline-induced fibrotic human gingival fibroblasts (HGFs) and reduced the expression of key fibrotic markers such as collagen type I, α-SMA, and TGF-β. These findings suggest that naringenin not only protects cells from cytotoxic damage but also exerts antifibrotic effects by modulating critical pathways like TGF-β, highlighting its therapeutic potential for fibrotic conditions such as oral submucous fibrosis (OSMF).

In alignment with previous studies, such as those by Mishra et al., which demonstrated increased collagen accumulation in advanced OSMF, using Masson's Trichrome (MT) staining, highlighting an increase in dense fibrotic tissue as the disease advances.^27^ Consistent with these findings, our study further confirms the histopathological changes in collagen organization across different stages of OSMF. Additionally, elastic fibers, which were initially dense in early-stage OSMF, became sparse in advanced stages. These alterations contribute to the characteristic fibrosis and trismus observed clinically. Furthermore, our findings on naringenin-treated fibroblasts provide a novel perspective, demonstrating a significant reduction in collagen accumulation under MT staining, resembling the morphology of control fibroblasts. This suggests that naringenin attenuates arecoline-induced fibrosis by modulating collagen synthesis, reinforcing its potential role as an anti-fibrotic agent in OSMF management.

The study by Belmina et al., on the in silico analysis of grapefruit seed extract components against SARS-CoV-2 main protease highlights molecular targets and pathways primarily associated with antiviral mechanisms. It identifies critical molecular interactions and pathways linked to viral inhibition, with a focus on disrupting SARS-CoV-2 replication by targeting the main protease. Key findings include the identification of bioactive components that interact with viral proteases, emphasizing their potential as antiviral agents.^28^ In contrast, in our study on naringenin's antifibrotic effects in oral submucous fibrosis (OSMF) focuses on molecular targets such as MMP2, MMP3, MMP9, TGFB1, ESR1, and SERPINE1, which are crucial in ECM remodeling and inflammation. As reported by Murugesan et al., SERPINE1 is upregulated in OSCC, and its inhibition may help prevent malignant transformation.^29^ The enrichment analyses reveal significant pathways like the PI3K-Akt signaling pathway, ECM-receptor interactions, and proteoglycans in cancer, highlighting their relevance in tissue fibrosis. While both studies leverage molecular docking and pathway analysis to identify therapeutic potentials, the naringenin study emphasizes antifibrotic mechanisms and tissue remodeling, whereas the grapefruit seed extract study focuses on antiviral mechanisms targeting viral proteases.

The findings of this study underscore the significant antifibrotic potential of naringenin in mitigating arecoline-induced fibrosis in human gingival fibroblasts, highlighting its therapeutic relevance for oral submucous fibrosis (OSMF). Cytological analyses using Masson's trichrome staining revealed a substantial reduction in collagen deposition following naringenin treatment, corroborating its ability to counteract fibrotic progression. Our study further confirmed that naringenin effectively downregulates key fibrotic markers, including collagen type I, α-SMA, and TGF-β, thereby modulating ECM remodeling. Additionally, pharmacological network analysis identified pivotal pathways, including TGF-β, PI3K-Akt, and MAPK signaling, as central to naringenin's antifibrotic mechanism, with hub genes such as MMP9 and TGFB1 playing critical roles.^26^, ^29^ These findings suggest that naringenin not only prevents excessive ECM deposition but also influences molecular pathways implicated in fibrosis progression. Furthermore, its ability to restore normal fibroblast morphology and improve cell viability against arecoline-induced cytotoxicity highlights its protective effects at the cellular level. Given its promising in vitro efficacy, naringenin presents a viable natural alternative for OSMF management, potentially complementing conventional antifibrotic therapies. However, further in vivo and clinical studies are imperative to validate its therapeutic potential, optimize dosing strategies, and assess long-term safety for clinical translation.

Limitations: Despite demonstrating promising antifibrotic effects of naringenin in vitro, this study has several limitations that should be acknowledged. Firstly, the experimental model is limited to cultured HGFs, which, while relevant to OSMF, do not fully replicate the complex tissue microenvironment, systemic influences, or multifactorial etiology of the disease. In vivo validation will be essential to confirm translational relevance. The gingival fibroblasts have inherent excellent healing properties, thus, may overestimate the efficacy of naringenin as antifibrotic agent. Secondly, the treatment duration was confined to short-term exposure (24–72 h), which may not accurately reflect the chronic and progressive nature of fibrosis. The absence of a positive control, such as a known clinically approved antifibrotic agent, restricts comparative evaluation of naringenin's therapeutic potential. Additionally, only a single concentration (40 μg/mL) was used in downstream analyses, limiting insights into its dose-dependent effects, therapeutic window, and safety margins. The reliance on molecular marker expression and histological assessments, without inclusion of functional assays like fibroblast contraction or migration, also narrows the scope of mechanistic understanding. Moreover, the fibrosis model, based solely on arecoline exposure, overlooks the multifactorial pathogenesis of OSMF, including contributions from copper ions, inflammatory mediators, and genetic factors. Further, pharmacokinetic and pharmacodynamic aspects were not addressed, leaving uncertainties about naringenin's bioavailability, metabolic stability, and systemic safety in physiological contexts. While GC-MS analysis confirmed naringenin as a major constituent of the grapefruit peel extract, the potential contribution of other bioactive compounds was not evaluated, which may confound attribution of effects solely to naringenin. Finally, detailed characterization of the fibroblast cell source, such as donor age, sex, or health status, was not assessed, which may influence cellular responses and limit the generalizability of the findings.

## 5 Conclusion

The present preliminary and exploratory study demonstrated the significant antifibrotic potential of naringenin in mitigating arecoline-induced fibrosis in human gingival fibroblasts. By effectively downregulating key fibrotic markers and modulating critical pathways such as TGF-β and PI3K-Akt, naringenin reduces collagen deposition and restores normal fibroblast morphology. The cross-validation between wet-lab and computational predictions in the present study adds robustness to our hypothesis formulation and promotes future translational studies. These findings highlight its potential as a therapeutic agent for OSMF, warranting further in vivo and clinical investigations to establish its efficacy and translational applications.

## Patient's/Guardian's consent

For invitro studies formal consent is not required.

## Patient's/Guardian's consent

For retrospective study formal consent is not required.

## Funding details

No funding was received for the research, authorship, and/or publication of this article.

## Declaration of financial and other interest

Nil.

## Ethical clearance

Approval was sought from the scientific review board (SRB/SDC/OPATH-2305/24/163) and Saveetha Dental College-Institutional Human Ethical Committee (IHEC/SDC/OPATH-2305/24/149)

## Ethical clearance

Ethical approval was granted by the Institutional Human Ethics Committee (IHEC/SDC/OPATH-2204/24/141).

## Sources of funding

The authors have not disclosed any funding.

## Declaration of competing interest

On behalf of all the authors I declare that we have no conflict of interest to declare.
